# Supplementary material for: Cosmetic makeup enhances facial attractiveness and affective neural responses
Source: PLoS One. 2022 Aug 15;17(8):e0272923. doi: 10.1371/journal.pone.0272923 (PMC9377592; doi:10.1371/journal.pone.0272923)
Supplement: Attachment — Submitted filename: Response to reviewers.docx [file pone.0272923.s001.docx]

**Point-by-point response to editor and reviewers’ comments:**

We thank the editor and reviewers for positively receiving our manuscript. We are grateful to receive constructive and useful suggestions. In the revised manuscript that we submit with this response letter, we have undertaken required changes to address all the comments made. All the changes in the revised manuscript (in reference to original submitted manuscript) are in red text. Below also find our point-by-point response to each comment.

**Journal Requirements:**

Comment 1:

**Please ensure that your manuscript meets PLOS ONE's style requirements, including those for file naming.**

Author response:

File names have been edited to comply with the style requirements.

Comment 2:

**As per the PLOS ONE policy (http://journals.plos.org/plosone/s/submission-guidelines#loc-human-subjects-research) on papers that include identifying, or potentially identifying, information, the individual(s) or parent(s)/guardian(s) must be informed of the terms of the PLOS open-access (CC-BY) license and provide specific permission for publication of these details under the terms of this license. Please download the Consent Form for Publication in a PLOS Journal (http://journals.plos.org/plosone/s/file?id=8ce6/plos-consent-form-english.pdf). The signed consent form should not be submitted with the manuscript, but should be securely filed in the individual's case notes. Please amend the methods section and ethics statement of the manuscript to explicitly state that the patient/participant has provided consent for publication: “The individual in this manuscript has given written informed consent (as outlined in PLOS consent form) to publish these case details”.**

Author response:

The written informed consents were obtained from all individuals who provided their facial photographs. In the revised manuscript, we have now added this information at relevant spaces (p. 12, line 261; p. 22, line 482).

Comment 3:

**In your Data Availability statement, you have not specified where the minimal data set underlying the results described in your manuscript can be found. PLOS defines a study's minimal data set as the underlying data used to reach the conclusions drawn in the manuscript and any additional data required to replicate the reported study findings in their entirety. All PLOS journals require that the minimal data set be made fully available. For more information about our data policy, please see http://journals.plos.org/plosone/s/data-availability.**

Author response:

We have uploaded the supporting information in a public repository (https://doi.org/10.6084/m9.figshare.16755682.v1). This information is added to the revised manuscript (p. 48, lines 1085–1086).

**Additional Editor Comments:**

**Although the study topic is enjoyable for both Reviewers, Reviewer#1 raised critical methodological issues that they must solve. First, the authors should connect the lacking Introduction assumptions and the Discussion. Reviewer#1 suggests that statistical analyses of the behavioral data should be re-performed, and authors should also provide a valuable Discussion of facial discrimination. Reviewerr#2 is more optimistic, although it raises several minor points that the authors should address. I am confident that the authors resolve all submitted comments, making them improve the quality of their paper.**

Author response:

We thank you for your positive comments. In the revised manuscript, we have addressed all the comments made by the reviewers; we agree that these suggestions have really helped in improving the quality of the paper.

**Reviewer #1 (Dr. Hirohito M. Kondo):**

Comment 1:

**The literature is reviewed in a clear and concise manner. However, the aim of the present study is vague. The authors examined whether makeup evokes greater attractiveness-related brain responses when presented with an individual’s own face than when presented with another person’s face (p. 7, line 151). It is unclear whether their assumptions regarding changes in brain responses depend on makeup effects, facial attractiveness, or discrimination between self and others (p. 9, line 184). The authors should clearly explain the relationship between task processes and ERP components.**

Author response:

We thank the reviewer for finding the literature clear and concise. To more clearly present our idea on the aim of this study, in the revised manuscript, we have provided a re-organized description on the research objectives and hypotheses in the introduction section (pp. 7–10, lines 145–214).

In this study, our primary goal was to explore the effects of makeup on ERP components elicited by facial images. Among the components, we were particularly interested in attractiveness-related ERP components (i.e., N170, EPN, P300, and LPP). Our secondary goal was to clarify whether these makeup effects occurred not only for others’ faces but also for one’s own face. For these purposes, we used two different types of classification tasks in which the participant had to look carefully at facial images: a task in which makeup was relevant (Experiment 1) or irrelevant (Experiment 2) to the task demand. Therefore, we were not particularly interested in the process of self–other face discrimination in this study.

Comment 2:

**In Experiment 1, the authors found that participants identified their own faces without makeup faster than they did with makeup. Participants rated their own made-up faces more attractive than without makeup. Previous studies have indicated that people detect attractive faces automatically and keep looking at them (p. 7, line 139). Are the present results consistent with previous findings? In addition, RTs derived from Experiment 2 were faster for participant’s own made-up faces than for their own faces without makeup. Thus, I wonder whether the behavioral results are reliable. It is well known that RT data do not follow a normal distribution. The authors should perform a logarithmic transformation of RT data (or use median RTs) and conduct the statistical analysis again.**

Author response:

We thank you for your insightful comments and suggestion. To address the RT distribution issue, we have re-conducted the statistical analysis and computed the median RT for each condition (p. 16, line 363; p. 17, Table 1). As a result, the difference between the makeup and no makeup condition failed to reach the significance level in Experiment 1 (p. 17, lines 374–377), although RTs were numerically longer for faces with makeup than those for faces without makeup. In Experiment 2, the median computation did not change the result: RTs were significantly shorter for faces with makeup than those for faces without makeup (p. 27, lines 578–579). This discrepancy between the two experiments can be explained by the relationship between the effects of makeup on facial appearance and the task demands. Makeup reduces facial distinctiveness by obscuring skin roughness and blotches, and makes faces look more feminine by increasing facial luminance and color contrast. In Experiment 1, the participant had to judge whether a face presented on the screen was her own face with or without makeup. In this task, faces without makeup were possibly easier to be discriminated than those with makeup because of the former’s greater distinctiveness, even though the RT difference did not reach the statistical significance. In Experiment 2, the participant had to judge whether a presented face was male or female. It is possible that increased facial femininity with makeup helped participants to classify faces as female in this task. In addition to the revised RT computation, we have also discussed these interpretations in the revised manuscript (pp. 34–35, lines 763–772).

Comment 3:

**Interpretations of results from Experiment 1 are not consistent. The authors stated that when participants viewed their own faces with makeup, larger EPN and LPP waveforms were elicited than when they viewed their own faces without makeup (p. 18, line 400). However, although the amplitude of the LPP component was greater with makeup, the amplitude of the EPN component was greater without makeup (Fig. 2). Do the amplitudes of ERP components reflect differences in facial attractiveness or RTs?**

Author response:

Larger EPN amplitudes indicate more negative voltages as the EPN is a negative-going deflection in the ERP waveform. We have now added this information in the revised manuscript (p. 18, lines 399–400) for clarity. Furthermore, we do not think that RT differences can explain the ERP differences between faces with and without makeup. This is because the effects of makeup on speed of face classification were different between the two experiments, and depended on the task demand. In contrast, ERP differences were constant in both the experiments. Therefore, we believe that these ERP differences reflect positive affective responses associated with perceived facial attractiveness. To increase clarity, we have now added this perspective in the general discussion section of the revised manuscript (pp. 34–35, lines 757–774).

Comment 4:

**For Experiment 2, why did the authors analyze the P200 component? The Introduction offers no explanation of mental processes based on P200. It is very confusing. If the P200 component is important in recognizing one’s face, it should be analyzed in Experiment 1 as well.**

Author response:

We thank you for your insightful comment. The occipitotemporal P200 component is known to be sensitive to self and other face discrimination, and hence, we have initially analyzed it in Experiment 2 only. However, in the revised manuscript, we first introduce the P200 component in the introduction section (p. 9, lines 197–204), and also determined it in Experiment 1 (p. 16, lines 347, 351 and 365; p. 18, lines 394–395).

Comment 5:

**The authors used different EEG setups in Experiments 1 and 2. Locations of electrodes also differed between the two experiments. Thus, the results of Experiment 1 are not directly comparable to those of Experiment 2. The authors should explain the reasons for these different EEG arrangements.**

Author response:

Due to laboratory constraints, we have employed different EEG recording systems in Experiments 1 and 2; however, both the EEG systems have been reliably used in many previous studies. We have now mentioned this point in the revised manuscript for clarity (p. 25, lines 549–551).

Comment 6:

**The authors reported significant differences in brain responses between experimental conditions, but the effect size in each case was very small. I am not sure that these results are robust. Are there any criteria regarding the effect size?**

Author response:

We thank you for your insightful comment. As described in the original manuscript, Cohen’s within-subject *d*z values for the makeup effect on EPN and LPP amplitudes were 0.465 and 0.486, respectively. To facilitate comparisons, we also calculated Cohen’s *dz* values for the makeup effect in Experiment 2 (p. 25, lines 564–565): 0.323 and 0.318 for EPN and LPP amplitudes, respectively (p. 30, lines 658–659; p. 31, lines 680–681). According to Cohen (1988)’s convention, these effect sizes can be regarded as small to medium. To increase clarity, we have now discussed the makeup effect size in the general discussion section of the revised manuscript (p. 33, lines 741–743).

Comment 7:

**My impression is that the manuscript lacks logical connections between the Introduction and the Discussion. First, what is the ecological relevance of distinguishing made-up faces of self from those of others? In daily life, we see other's faces, but we rarely see our own. I think that makeup can change one’s mood (or increase extroversion along with self-confidence). Do such changes in mood affect brain responses? Makeup can also enhance the salience of facial features (luminance, color, contrast, etc.). The authors discuss effects of makeup on facial attractiveness and visual attention, but not the effect of salience on object detection. They should clearly explain how ERP components are associated with mental processes of face recognition.**

Author response:

We thank you for your insightful comments. As described above (response to comment 1), we chose two classification tasks in which makeup was relevant or irrelevant to the task demand. Although not related to real-world, these tasks made participants look carefully at facial images. Regarding the mood effect, we do not think that it was related to the ERP enhancement because the effect of makeup occurred even when participants wore no makeup during the face categorization task in Experiment 1. However, future studies should answer the question on whether makeup-induced mood changes lead to further enhancement of the ERP effect. Regarding facial features saliency, we did not deal with early visual components like P100, which reflect visual processing of low-level facial properties such as local luminance and contrast or spatial frequency. Two previous ERP studies examined the effect of makeup on P100 amplitudes; however, both studies failed to find a statistically significant result (Tagai et al., 2017; Tanaka, 2016). Therefore, we focused on attractiveness-related ERP components, ranging from N170 indexing configural processing of faces to EPN, P300, and LPP reflecting automatic or motivated attentional allocation to affective meanings of faces. We added this description in the introduction of the revised manuscript (p. 7, lines 146–148).

Comment 8:

**I cannot read the descriptions in the figures (e.g., Figs 2 and 4) because of low resolution. Please improve the quality of figures.**

Author response:

We thank you for your comment. Figures in a merged PDF will almost certainly look blurry. We provide high resolution figures during the submission of the revised manuscript.

**Reviewer #2 (Dr. Caitlin A. J. Powell):**

Comment 1:

**On the whole, this study has strengths that are worth mentioning -- I have a few clarification points that I would like to see, however.
First of all, the literature review is thorough, and the novelty of the research is clear in terms of how it expands on the literature. I appreciated the expansion of the research to focus on reactions to one's own face, to comparing one's own face with another's, and the exploration of whether the results would still occur under implicit conditions. Likewise, having the more realistic notion of makeup that has been self-applied has its benefits, although it also provided some downsides, which I'll expand on below. The description of the methodology used was thorough, and the sample size was sufficient for research of this type, and for the most part, I found the analysis to likewise be complete and comprehensible. The discussion was clear at highlighting the key findings, and was reasonably transparent in terms of methodological challenges (i.e having them apply their own makeup without strict consistency).**

Author response:

We thank the reviewer for encouraging and positive comments on our manuscript.

Comment 2:

**There were a few things I would like to see added to the study:**

**1) The description of the makeup the participants were told to apply was 'makeup that they would normally wear while going out with their friends on the weekend.' (p. 12, ln. 251-252, also in Study 2 on p. 22, ln 491-492). On page 19, ln. 413, the makeup worn is classified as 'ordinary makeup'. This is not day-to-day wear -- it is makeup explicitly worn only on the weekends for a night out. This, to a certain extent, could help explain some of the results in terms of reaction time, as participants would be more used to seeing their bare faces, or the makeup they may have worn while at work, as opposed to the rarer, dressier makeup they were asked to put on. The researchers should further clarify how the type of makeup could differ from daily wear, and not refer to it as 'ordinary' makeup, as that could be confusing.**

Author response:

In the revised manuscript, we have replaced the phrase “ordinary makeup” with “self-applied makeup” (p. 8, line 175; p. 10, line 205; p. 19, line 425; p. 20, line 431; p. 33, line 727). In addition, we have added a description on the limitation of the style of self-applied makeup we used in our study in the general discussion section (p. 37, lines 826–829).

Comment 3:

**2) While the effect sizes were appropriate to the type of test used, the authors should acknowledge effect size in their discussion of results, making sure to specify not only a significant finding, but clarifying when a result was a large or more modest effect to better contextualize the results -- as well as briefly identifying what is a typical effect size for studies using this methodology.**

Author response:

Across the two experiments, the effect size (within-subject Cohen’s *d*z) of makeup on ERP amplitudes was 0.3–0.4, which can be regarded as small to medium according to Cohen (1988)’s convention. We added this information in the general discussion section of the revised manuscript (p. 33, lines 741–743).

Comment 4:

**3) The authors report all cell means and standard deviations, but in Study 2, there are several main effect tests that are significant; providing the main effect means and standard deviations would be preferable in addition to cell means. (p.27, lns 572, 573, 579, 589 -- or added to Table 2; p. 29 ln 627 and 635; p. 30 ln 644, 651, 652).**

Author response:

We computed the main effect means and standard deviations for the makeup factor, and reported them in the body of manuscript and Table of the revised manuscript (p. 26, Table 2; p. 29, lines 636–638 and 646–648; p. 30, lines 656–658 and 667–669; p. 31, lines 678–680).

**References**

Cohen, J. (1988). *Statistical power analysis for the behavioral sciences*. Routledge.

Tagai, K., Shimakura, H., Isobe, H., & Nittono, H. (2017). The light-makeup advantage in facial processing: Evidence from event-related potentials. *PLoS One*, *12*(2), e0172489. doi: 10.1371/journal.pone.0172489.

Tanaka, H. (2016). Facial cosmetics exert a greater influence on processing of the mouth relative to the eyes: Evidence from N170 event-related potential component. *Frontiers in psychology*, *7*, 1359. doi: 10.3389/fpsyg.2016.01359.
