# Supplementary material for: Cosmetic makeup enhances facial attractiveness and affective neural responses
Source: PLoS One. 2022 Aug 15;17(8):e0272923. doi: 10.1371/journal.pone.0272923 (PMC9377592; doi:10.1371/journal.pone.0272923)
Supplement: Attachment — Submitted filename: Response to Reviewers.docx [file pone.0272923.s002.docx]

**Point-by-point response to editor and reviewers’ comments:**

We thank the editor and reviewers for positively receiving our manuscript. We are grateful to receive constructive and useful suggestions. In the revised manuscript that we submit with this response letter, we have undertaken required changes to address all the comments made. All the changes are highlighted in the file labeled “Revised Manuscript with Track Changes.” Below also find our point-by-point response to each comment.

**Journal Requirements:**

Comment 1:

**Please review your reference list to ensure that it is complete and correct. If you have cited papers that have been retracted, please include the rationale for doing so in the manuscript text, or remove these references and replace them with relevant current references. Any changes to the reference list should be mentioned in the rebuttal letter that accompanies your revised manuscript. If you need to cite a retracted article, indicate the article’s retracted status in the References list and also include a citation and full reference for the retraction notice.**

Author response:

We have reviewed our reference list and found that it is complete and correct. No retracted papers have been cited. There are no changes to the reference list.

**Additional Editor Comments:**

**I am very sorry for the long delay. I have found it seriously difficult to find expert reviewers. However, I think the paper still needs a minor revision at this stage. Given the long delay, I suggest the authors revise their manuscript by solving the reviewer comments and then submit their last revision for acceptance. Please accompany the revised manuscript with a detailed letter explaining how you have responded to each of the reviewers' points, and where the changes appear in the revised manuscript.**

Author response:

We thank you for everything in reviewing our manuscript. We hope that the revised manuscript now meets the standards of the journal.

**Reviewer #1:**

Comment 1:

**The several descriptions remain difficult to understand. I pointed them out in my previous comments, but the authors did not update their manuscript. For example, in lines 397-399, the authors stated that “Viewing one’s own face with makeup on resulted in larger EPN waveforms than viewing one’s own face without makeup (makeup: M = 2.60 μV, SD = 2.62 μV; no makeup: M = 2.92 μV, SD = 2.44 μV).” What does a larger waveform mean? That statement does not depend on whether the waveform indicates positive or negative deflection. The authors also stated that “Another key finding was that faces with makeup elicited larger EPN and LPP amplitudes regardless of whether the presence of makeup was explicitly relevant to the participant’s task" (lines 781-783). This is misleading. As described above, the amplitude of the EPN component was larger for the non-makeup condition. I found that the EPN component was negatively biased for the makeup condition than for non-makeup condition, whereas the LPP component was positively biased for the makeup condition than for non-makeup condition. The authors should check their statements in all the sections and reword them appropriately.**

Author response:

We thank you for your helpful comments. To clarify the magnitude of voltage deflections in each ERP component, we have replaced all phrases “larger” with “more negative” or “more positive” depending on its polarity (line 25, line 105, line 107, line 114, lines 127–129, lines 217–223, lines 414–417, line 425, lines 440–443, line 454, line 459, line 480, line 661, line 663, lines 676–677, line 686, line 687, line 701, line 702, line 712, line 713, line 727, line 733, line 740, line 757, lines 763–765, line 770, lines 795–796, line 806, line 809, lines 816–817, line 835, lines 845–846, line 865, and line 900). We think that the current descriptions are more objective and straightforward than before.

Comment 2:

**Technical comment: the authors should enlarge the font size in the figures.**

Author response:

We thank you for your comment. We have increased the font sizes of texts in Figure 2 and Figure 4 for better readability.
